# Involvement of RhoA-mediated Ca^2+ ^sensitization in antigen-induced bronchial smooth muscle hyperresponsiveness in mice

**DOI:** 10.1186/1465-9921-6-4

**Published:** 2005-01-08

**Authors:** Yoshihiko Chiba, Ayako Ueno, Koji Shinozaki, Hisao Takeyama, Shuji Nakazawa, Hiroyasu Sakai, Miwa Misawa

**Affiliations:** 1 Department of Pharmacology, School of Pharmacy, Hoshi University, 2-4-41 Ebara, Shinagawa-ku, Tokyo 142-8501, Japan

## Abstract

**Background:**

It has recently been suggested that RhoA plays an important role in the enhancement of the Ca^2+ ^sensitization of smooth muscle contraction. In the present study, a participation of RhoA-mediated Ca^2+ ^sensitization in the augmented bronchial smooth muscle (BSM) contraction in a murine model of allergic asthma was examined.

**Methods:**

Ovalbumin (OA)-sensitized BALB/c mice were repeatedly challenged with aerosolized OA and sacrificed 24 hours after the last antigen challenge. The contractility and RhoA protein expression of BSMs were measured by organ-bath technique and immunoblotting, respectively.

**Results:**

Repeated OA challenge to sensitized mice caused a BSM hyperresponsiveness to acetylcholine (ACh), but not to high K^+^-depolarization. In α-toxin-permeabilized BSMs, ACh induced a Ca^2+ ^sensitization of contraction, which is sensitive to *Clostridium botulinum *C3 exoenzyme, indicating that RhoA is implicated in this Ca^2+ ^sensitization. Interestingly, the ACh-induced, RhoA-mediated Ca^2+ ^sensitization was significantly augmented in permeabilized BSMs of OA-challenged mice. Moreover, protein expression of RhoA was significantly increased in the hyperresponsive BSMs.

**Conclusion:**

These findings suggest that the augmentation of Ca^2+ ^sensitizing effect, probably via an up-regulation of RhoA protein, might be involved in the enhanced BSM contraction in antigen-induced airway hyperresponsiveness.

## Background

Increased airway narrowing in response to nonspecific stimuli is a characteristic feature of human obstructive diseases, including bronchial asthma. This abnormality is an important symptom of the disease, although the pathophysiological variations leading to the hyperresponsiveness are unclear now. Several mechanisms have been suggested to explain the airway hyperresponsiveness (AHR), such as alterations in the neural control of airway smooth muscle [1], increased mucosal secretions [2], and mechanical factors related to remodeling of the airways [3]. In addition, it has also been suggested that one of the factors that contribute to the exaggerated airway narrowing in asthmatics is an abnormality of the nature of airway smooth muscle [4,5]. Rapid relief from airway limitation in asthmatic patients by β-stimulant inhalation may also suggest an involvement of augmented airway smooth muscle contraction in the airway obstruction. Thus, it may be important for development of asthma therapy to understand changes in the contractile signaling of airway smooth muscle cells associated with the disease.

Smooth muscle contraction including airways is mainly regulated by an increase in cytosolic Ca^2+ ^concentration in myocytes. Recently, additional mechanisms have been suggested in agonist-induced smooth muscle contraction by studies in which the simultaneous measurements of force development and intracellular Ca^2+ ^concentration, and chemically permeabilized preparations in various types of smooth muscles were used. It has been demonstrated that agonist stimulation increases myofilament Ca^2+ ^sensitivity in permeabilized smooth muscles of the rat coronary artery [6], guinea pig vas deferens [7], canine trachea [8] and rat bronchus [9]. Although the detailed mechanism is not fully understood, a participation of RhoA, a monomeric GTP binding protein, in the agonist-induced Ca^2+ ^sensitization has been suggested by many investigators [10]. Moreover, an augmented RhoA-mediated Ca^2+ ^sensitization in smooth muscle contraction has been reported in experimental animal models of diseases such as hypertension [11-13], coronary [14-16] and cerebral [17-19] vasospasm. It is thus possible that RhoA-mediated signaling is the key for understanding the abnormal contraction of diseased smooth muscles.

Here, we show an increased acetylcholine (ACh)-induced contraction of bronchial smooth muscle (BSM) isolated from repeatedly ovalbumin (OA)-challenged BALB/c mice, which have been reported to have in vivo AHR [20]. A participation of RhoA-mediated Ca^2+ ^sensitization in the augmented ACh-induced contraction of BSM was demonstrated in this animal model of AHR.

## Methods

### Sensitization and antigenic challenge

Male BALB/c mice (6-week old, specific pathogen-free; Charles River Japan, Inc., Kanagawa, Japan) were used. All experiments were approved by the Animal Care Committee at the Hoshi University (Tokyo, Japan). Preparation of a murine model of allergic bronchial asthma, which has in vivo airway hyperresponsiveness (AHR), was performed as described by Kato *et al*. [20]. In brief, mice were actively sensitized by intraperitoneal injections of 8 μg ovalbumin (OA; Seikagaku Co., Tokyo, Japan) with 2 mg Imject Alum (Pierce Biotechnology, Inc., Rockfold, IL, USA) on day 0 and day 5. The sensitized mice were challenged with aerosolized OA-saline solution (5 mg/ml) for 30 min on days 12, 16 and 20. A control group of mice received the same immunization procedure but inhaled saline aerosol instead of OA challenge. The aerosol was generated with an ultrasonic nebulizer (Nihon Kohden, Tokyo, Japan) and introduced to a Plexiglas chamber box (130 × 200 mm, 100 mm height) in which the mice were placed.

### Determination of intact bronchial smooth muscle (BSM) responsiveness

Twenty-four h after the last antigen challenge, the mice were sacrificed by exsanguination from abdominal aorta under urethane (1.6 g/kg, *i.p*.) anesthesia. Then the airway tissues under the larynx to lungs were immediately removed. About 3 mm length of the left main bronchus (about 0.5 mm diameter) was isolated and epithelium was removed by gently rubbing with keen-edged tweezers [21]. The resultant tissue ring preparation was then suspended in a 5 ml-organ bath by two stainless-steel wires (0.2 mm diameter) passed through the lumen. For all tissues, one end was fixed to the bottom of the organ bath while the other was connected to a force-displacement transducer (TB-612T, Nihon Kohden) for the measurement of isometric force. A resting tension of 0.5 g was applied. The buffer solution contained modified Krebs-Henseleit solution with the following composition (mM); NaCl 118.0, KCl 4.7, CaCl_2 _2.5, MgSO_4 _1.2, NaHCO_3 _25.0, KH_2_PO_4 _1.2 and glucose 10.0. The buffer solution was maintained at 37°C and oxygenated with 95% O_2_-5% CO_2_. The BSM responsiveness to exogenously applied Ca^2+ ^in acetylcholine (ACh)-stimulated or high K^+^-depolarized muscle was determined as previously [22]. In brief, after an equilibration period, the organ bath solution was replaced with Ca^2+^-free solution containing 10^-6 ^M nicardipine with the following composition (mM); NaCl 122.4, KCl 4.7, MgSO_4 _1.2, NaHCO_3 _25.0, KH_2_PO_4 _1.2, glucose 10.0 and EGTA 0.05. Fifteen min later, 1 mM ACh was added and, after attainment of a plateau (almost baseline level) response to ACh, a cumulative concentration-response curve for Ca^2+ ^(0.1–6.0 mM) was made. A higher concentration of Ca^2+ ^was added after the response to the previous concentration reached a plateau. In another series of experiments, bronchial smooth muscles were depolarized with 60 mM K^+^, instead of ACh, in the presence of 10^-6 ^M atropine and in the absence of nicardipine in the Ca^2+^-free solution. All these functional studies were performed in the presence of 10^-6 ^M indomethacin. The concentration of indomethacin had no effect both on baseline tension and on the ACh- and high K^+^-induced constrictions of BSMs (data not shown).

### BSM permeabilized fiber experiments

To determine the change in Ca^2+ ^sensitization of BSM contraction, permeabilized BSMs were prepared as described previously [21] with minor modification. In brief, 24 h after the last antigen challenge, the left main bronchus was isolated as described above and cut into ring strips (about 200 μm width, 500 μm diameter). The epithelium was removed by gently rubbing with keen-edged tweezers. The ring strips were then permeabilized by a 30-min treatment with 83.3 μg/ml α-toxin (Sigma, St. Louis, MO, USA) in the presence of Ca^2+ ^ionophore A23187 (10 μM, Sigma) at room temperature in relaxing solution. Relaxing solution contained: 20 mM PIPES, 7.1 mM Mg^2+^-dimethanesulfonate, 108 mM K^+^-methanesulfonate, 2 mM EGTA, 5.875 mM Na_2_ATP, 2 mM creatine phosphate, 4 U/ml creatine phosphokinase, 1 μM carbonyl cyanide p-trifluoromethoxyphenylhydrazone (FCCP) and 1 μg/ml E-64 (pH 6.8) containing 10 μM A23187. Free Ca^2+ ^concentration was changed by adding an appropriate amount of CaCl_2_. The apparent binding constant of EGTA for Ca^2+ ^was considered to be 10^6 ^M^-1 ^[23]. The permeabilized muscle strip was then suspended in a 400-μL organ bath at room temperature. The contractile force developed was measured by an isometric transducer (T7-8-240; Orientec, Tokyo, Japan) under a resting tension of 50 mg. To determine the involvement of RhoA in the ACh-induced myofilament Ca^2+ ^sensitization, the α-toxin-permeabilized muscle strips were treated with *Clostridium botulinum *C3 exoenzyme (10 μg/ml; Calbiochem-Novabiochem Corp., La Jolla, CA) in the presence of 100 μM NAD for 20 min at room temperature.

### Determination of RhoA protein level in BSM

Protein samples of BSMs were prepared as previously [21]. In breif, the airway tissues below the main bronchi to lungs were removed and immediately soaked in ice-cold, oxygenated Krebs-Henseleit solution. The airways were carefully cleaned of adhering connective tissues, blood vessels and lung parenchyma under a stereomicroscopy. The epithelium was removed as much as possible by gently rubbing with keen-edged tweezers [21]. Then the bronchial tissue (containing the main and intrapulmonary bronchi) segments were quickly frozen with liquid nitrogen, and the tissue was crushed to pieces by CryopressTM (CP-100W; Microtec, Co. Ltd., Chiba, Japan: 15 sec × 3). The tissue powder was homogenized in ice-cold tris(hydroxymethyl)aminomethane (Tris, 10 mM; pH 7.5) buffer containing 5 mM MgCl_2_, 2 mM EGTA, 250 mM sucrose, 1 mM dithiothreitol, 1 mM 4-(2-aminoethyl)benzenesulfonyl fluoride, 20 μg/ml leupeptin, 20 μg/ml aprotinin, 1% Triton X-100 and 1% sodium cholate. The tissue homogenate was then centrifuged (3,000 g, 4°C for 15 min) and the resultant supernatant was stored at -85°C until use. To determine the level of RhoA protein in BSMs, the samples (10 μg of total protein per lane) were subjected to 15% SDS-PAGE and the proteins were then electrophoretically transferred to a PVDF membrane. After blocking with 3% gelatin, the PVDF membrane was incubated with polyclonal rabbit anti-RhoA antibody (1:3,000; Santa Cruz Biotechnology, Inc., Santa Cruz, CA, USA). Then the membrane was incubated with horseradish peroxidase-conjugated goat anti-rabbit IgG (1:2,500 dilution; Amersham Biosciences, Co., Piscataway, NJ, USA), detected by an enhanced chemiluminescent system (Amersham Biosciences, Co.) and analyzed by a densitometry system. Thereafter, the primary and secondary antibodies were stripped and the membrane was reprobed by using monoclonal mouse anti-glyceraldehyde-3-phosphate dehydrogenase (GAPDH; 1:3,000 dilution; Chemicon International, Inc., Temecula, CA, USA) to confirm the same amount of proteins loaded.

### Determination of active form of RhoA in BSM

The active form of RhoA, GTP-bound RhoA, in BSMs was measured by RhoA pull down assay. In brief, bronchial tissues containing the main and intrapulmonary bronchi were isolated as described above. The isolated bronchial tissues were equilibrated in oxygenated Krebs-Henseleit solution at 37°C for 1 hr. After the equilibration period, the tissues were stimulated by ACh (10^-3 ^M for 10 min) and were quickly frozen with liquid nitrogen. The tissues were then lysed in lysis buffer with the following composition (mM); HEPES 25.0 (pH 7.5), NaCl 150, IGEPAL CA-630 1%, MgCl_2 _10.0, EDTA 1.0, glycerol 10%, NaF 25.0, sodium orthovanadate 1.0 and peptidase inhibitors. Active RhoA in tissue lysates (200 μg protein) was precipitated with 25 μg GST-tagged Rho binding domain (amino acids residues 7–89 of mouse rhotekin; Upstate, Lake Placid, NY, USA), which was expressed in *Escherichia coli *and bound to glutathione-agarose beads. The precipitates were washed three times in lysis buffer, and after adding the SDS loading buffer and boiling for 5 min, the bound proteins were resolved in 15% polyacrylamide gels, transferred to nitrocellulose membranes, and immunoblotted with anti-RhoA antibody as described above.

### Determination of phosphorylation of myosin phosphatase and myosin light chain in BSM

Phosphorylated proteins were detected by using the fluorescent Pro-Q-Diamond dye (Molecular Probes, Eugene, OR, USA), which can directly detect phosphate groups attached to tyrosine, serine or threonine residues in gels [24]. In brief, bronchial tissue lysates (50 μg protein) with SDS loading buffer prepared as described above were resolved in 10 – 20% gradient polyacrylamid gels (Atto Co., Tokyo, Japan). Proteins were fluorescently stained by fixing the gels in 50% methanol and 10% acetic acid for 1 h. The gels were washed with deionised water for 20 min, stained with Pro-Q-Diamond for 1.5 h and destained by three washes in 4% acetonitrile in 50 mM sodium acetate, pH 4.0, for 2 h. Gels were scanned with a fluorimager, a Typhoon 9410 laser scanner (Amersham Biosciences, Co.), with excitation at 532 nm and a 580 nm band pass emission filter for Pro-Q-diamond dye detection. Phosphorylated proteins were quantified densitometrically with the ImageQuant software (Amersham Biosciences, Co.). After scanning, the gels were washed with deionised water for 30 min and incubated in 0.7% glycine-0.2% SDS in 0.3% Tris buffer for 15 min. The proteins were then electrophoretically transferred to a PVDF membrane and immunoblottings for myosin phosphatase target subunit 1 (MYPT1; polyclonal goat anti-MYPT1 antibody; 1:1000; Santa Cruz Biotechnology, Inc.), GAPDH and myosin light chain (MLC; polyclonal rabbit anti-MLC2 antibody; 1:3000; Santa Cruz Biotechnology, Inc) were performed as described above.

### Statistical analyses

All the data are expressed as the mean ± S.E. Statistical significance of difference was determined by unpaired Student's *t*-test, Bonferroni/Dunn's test or two-way analysis of variance (ANOVA).

## Results

### Contractile response of intact BSM preparations

Under Ca^2+^-free condition (in the presence of 10^-6 ^M nicardipine and 0.05 mM EGTA), ACh (10^-3 ^M) generated a transient phasic contraction in all BSM preparations used. The generated tension of BSM from the repeatedly OA-challenged mice (69 ± 12 mg, N = 6) was significantly greater than that from the sensitized control animals (20 ± 12 mg, N = 6; P < 0.05). The concentration of nicardipine used in the present study completely blocked high K^+ ^(10–90 mM)-induced BSM contraction in Ca^2+ ^(2.5 mM) containing normal Krebs-Henseleit solution (data not shown), indicating that voltage-dependent Ca^2+ ^channels were completely blocked in this condition.

**Figure 1:**
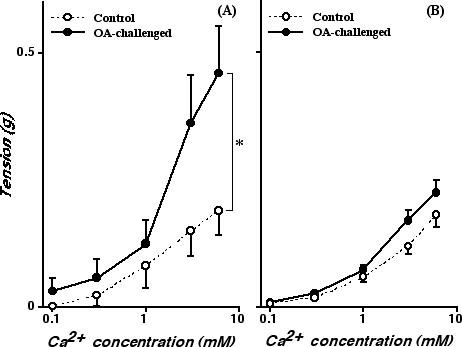
Cumulative concentration-response curves to Ca^2+ ^of bronchial rings obtained from sensitized control (Control; *open circles*) and repeatedly ovalbumin-challenged (OA-challenged; *closed circles*) mice. Bronchial rings were preincubated with 10^-3 ^M acetylcholine (ACh) in the presence of 10^-6 ^M nicardipine (*A*) or isotonic 60 mM K^+ ^in the presence of 10^-6 ^M atropine (*B*) in Ca^2+^-free, 0.05 mM EGTA solution. Each point represents the mean ± S.E. from 6 experiments. The Ca^2+^-induced contraction of the ACh-stimulated bronchial smooth muscles was significantly augmented in the OA-challenged group (*A*; P < 0.05 by ANOVA), whereas no significant change in the Ca^2+^-induced contraction of the high K^+^-depolarized muscles was observed between groups (*B*).

The tension returned to baseline level within 5 min after the ACh application, and then the contraction induced by cumulatively administered Ca^2+ ^was measured. Figure 1A shows the concentration-response curves to Ca^2+ ^of murine BSMs that were preincubated with nicardipine (10^-6 ^M) and ACh (10^-3 ^M) under Ca^2+^-free (0.05 mM EGTA) condition. Addition of Ca^2+ ^induced a concentration-dependent BSM contraction in both the sensitized control and OA-challenged groups. The contractile response to Ca^2+ ^of the ACh-stimulated BSMs from the repeatedly OA-challenged mice was markedly augmented as compared to that from the sensitized control animals. By contrast, no significant difference in the response to Ca^2+ ^of BSMs depolarized with 60 mM K^+ ^(in the absence of nicardipine and presence of 10^-6 ^M atropine) was observed between groups (Fig. 1B). Likewise, the ACh (10^-7^–10^-3 ^M) concentration-response curve determined in normal Krebs-Henseleit solution (2.5 mM Ca^2+^) was significantly shifted upward in BSMs from the OA-challenged mice as compared with that from the sensitized control animals, whereas no significant difference in the contractile response induced by isotonic high K^+ ^(10–90 mM) was observed between groups (data not shown).

### Contractile response of α-toxin-permeabilized BSM preparations

**Figure 2:**
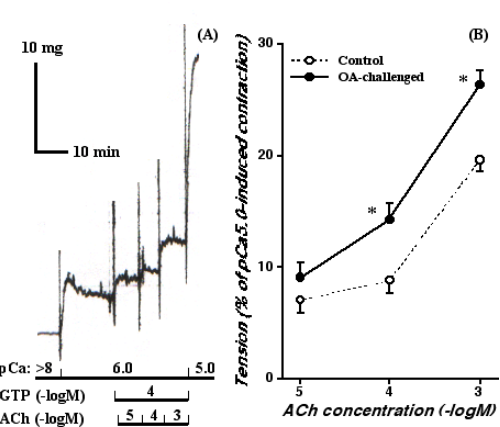
Acetylcholine (ACh)-induced Ca^2+ ^sensitization of murine bronchial smooth muscle. (*A*) A typical recording of contraction induced by Ca^2+ ^(pCa 6.0 and 5.0) and ACh (10^-5^–10^-3 ^M) with guanosine triphosphate (GTP; 10^-4 ^M) in α-toxin-permeabilized bronchial smooth muscle isolated from a sensitized control mouse. In the presence of GTP, ACh induced further contractions even in the constant Ca^2+ ^concentration at pCa 6.0, *i.e*., ACh-induced Ca^2+ ^sensitization, in an ACh-concentration-dependent manner. (*B*) Concentration-response curves for ACh (10^-5^–10^-3 ^M)-induced Ca^2+ ^sensitization in α-toxin-permeabilized bronchial smooth muscle isolated from sensitized control (Control; *open circles*) and repeatedly ovalbumin-challenged (OA-challenged; *closed circles*) mice. The data are expressed as percentage increase in tension induced by ACh (10^-5^–10^-3 ^M) in the presence of Ca^2+ ^(pCa 6.0) and GTP (10^-4 ^M) from the sustained contraction induced by pCa 6.0. Each point represents the mean ± S.E. from 6 experiments. The ACh-induced Ca^2+ ^sensitization of bronchial smooth muscle contraction was significantly augmented in the OA-challenged mice (*P < 0.05 vs. Control group by unpaired Student's *t*-test).

The BSM contractility was also determined by using α-toxin-permeabilized BSM preparations. In all BSM preparations treated with a-toxin, application of free Ca^2+ ^(pCa = 6.5, 6.3, 6.0, 5.5 and 5.0) induced a concentration-dependent reproducible contractile response, indicating successful permeabilization. In the α-toxin-permeabilized BSM, no significant difference in the Ca^2+ ^responsiveness or the maximal contractile response induced by pCa 5.0 (Emax) was observed between the sensitized control (pEC_50_[Ca^2+ ^(M)] = 5.67 ± 0.04, Emax = 26.7 ± 1.2 mg; N = 6) and repeatedly OA-challenged (pEC_50_[Ca^2+ ^(M)] = 5.78 ± 0.15, Emax = 22.8 ± 5.9 mg; N = 6) groups. In both groups, when the Ca^2+ ^concentration was clamped at pCa 6.0, application of ACh (10^-5^–10^-3 ^M) in the presence of GTP (10^-4 ^M) caused a further contraction, *i.e*., ACh-induced Ca^2+ ^sensitization, in an ACh concentration-dependent manner (Fig. 2). The ACh-induced Ca^2+ ^sensitization was significantly greater in the repeatedly OA-challenged group (Fig. 2B).

**Figure 3:**
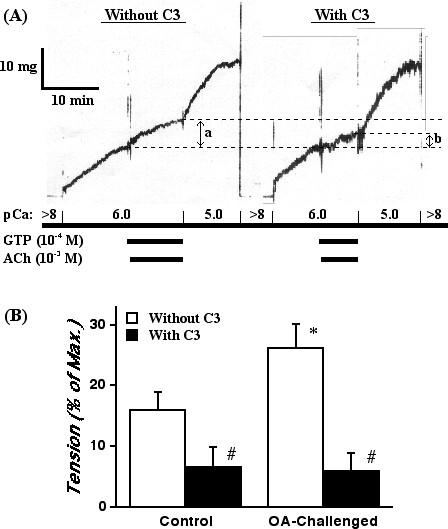
Effect of *Clostridium botulinum *C3 exoenzyme, an inhibitor of RhoA protein, on the acetylcholine (ACh)-induced Ca^2+ ^sensitization of the α-toxin-permeabilized bronchial smooth muscle of mice. (*A*) Typical recordings of contraction induced by Ca^2+ ^(pCa 6.0 and 5.0) and ACh (10^-3 ^M) with guanosine triphosphate (GTP; 10^-4 ^M) in α-toxin-permeabilized bronchial smooth muscle isolated from a sensitized control mouse. In the presence of GTP, ACh induced a further contraction even in the constant Ca^2+ ^concentration at pCa 6.0, *i.e*., ACh-induced Ca^2+ ^sensitization (*a*). The ACh-induced Ca^2+ ^sensitization was re-estimated after treatment with C3 exoenzyme (10 μg/mL, for 20 min; *b*). (*B*) Summary of the effects of C3 exoenzyme on the ACh-induced Ca^2+ ^sensitization of bronchial smooth muscle contraction in the sensitized control (Control) and repeatedly ovalbumin (OA)-challenged (OA-challenged) mice. The data are expressed as percentage increase in tension induced by ACh (in the presence of Ca^2+ ^and GTP) from the sustained contraction induced by pCa 6.0. Each column represents the mean ± S.E. from 6 experiments. *P < 0.05 vs. Control group (Before C3) and #P < 0.05 vs. respective Before C3 group by Bonferroni/Dunn's test.

To determine an involvement of RhoA protein in the ACh-induced Ca^2+ ^sensitization, the effect of pretreatment with C3 exoenzyme on the contractile response of the α-toxin-permeabilized BMS was also investigated. The C3 treatment alone had no significant effect on the Ca^2+ ^responsiveness of α-toxin-permeabilized BSMs in any groups (data not shown). However, the ACh (10^-3 ^M, in the presence of 10^-4 ^M GTP)-induced Ca^2+ ^sensitizing effect was inhibited by treatment with C3 in both the sensitized control and OA-challenged groups (Fig. 3). Interestingly, the remaining C3-insensitive component of the ACh-induced Ca^2+ ^sensitization was the same level between groups, whereas the Ca^2+ ^sensitization before treatment with C3 was significantly greater in BSMs of the OA-challenged mice (Fig. 3B). These findings indicate that the C3-sensitive Ca^2+ ^sensitization, probably mediated by RhoA [25,26], might be augmented in BSMs of the OA-challenged AHR mice.

### Upregulation of RhoA protein in BSMs of OA-challenged mice

**Figure 4:**
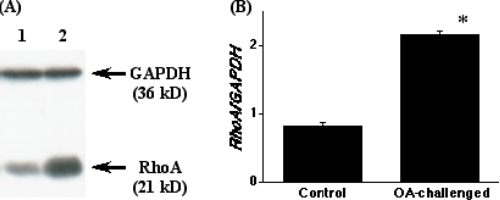
The levels of RhoA protein in the bronchi obtained from the sensitized control (Control) and repeatedly ovalbumin (OA)-challenged (OA-challenged) mice. (*A*) Typical immunoblots. *Lane 1*; Control, *lane 2*; OA-challenged, and GAPDH; glyceraldehyde-3-phosphate dehydrogenase. The bands were analyzed by a densitometer and normalized by the intensity of corresponding GAPDH band, and the data are summarized in *B*. Each column represents the mean ± S.E. from 5 experiments. The expression level of RhoA protein in the bronchi was significantly increased in the OA-challenged group (*P < 0.001 vs. Control group by unpaired Student's *t*-test).

**Figure 5:**
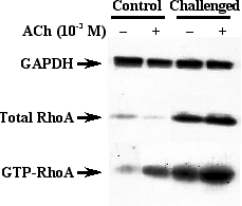
Representative immunoblots showing activation of RhoA in acetylcholine (ACh)-stimulated bronchi obtained from the sensitized control (Control) and repeatedly ovalbumin (OA)-challenged (Challenged) mice. Isolated bronchial tissues were incubated for 10 min in the absence (-) or presence (+) of 10^-3 ^M ACh (*see Methods*). Tissues were then rapidly lysed, GTP-bound active form of RhoA was pulled down with GST-tagged Rho binding domain of rhotekin, and RhoA was visualized by Western blotting. The respective blot of total RhoA in each sample is also shown. The GTP-bound RhoA in ACh-stimulated bronchi was markedly increased in the OA-challenged mice.

The expression of RhoA protein in BSM homogenates was assessed by using immunoblotting. As shown in Fig. 4A, immunoblotting with the antibody against RhoA gave a single 21 kD band, indicating the expression of RhoA protein in murine BSM. The level of RhoA protein in samples of the OA-challenged mice was significantly increased as compared with that of the sensitized control animals. Moreover, the GTP-bound active form of RhoA in ACh-stimulated BSMs was markedly increased in the OA-challenged mice (Fig. 5).

### Augmented ACh-induced phosphorylation of MLC in BSMs of OA-challenged mice

**Figure 6:**
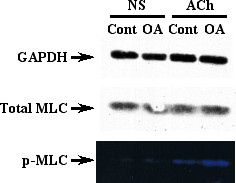
Representative photographs showing phosphorylation of myosin light chain (MLC) in acetylcholine (ACh)-stimulated bronchi obtained from the sensitized control (Cont) and repeatedly ovalbumin-challenged (OA) mice. Isolated bronchial tissues were incubated for 10 min in the absence (non-stimulated; NS) or presence of 10^-3 ^M ACh (*see Methods*). The electrophoretically separated proteins on gels were stained by Pro-Q Diamond dye, which can detect phosphorylated proteins specifically and quantitatively. After detection of phosphorylated proteins, immunoblotting for MLC was performed to detect total (phosphorylated and non-phosphorylated) MLC. The respective Pro-Q Diamond dye-positive band (*bottom panel*), which has same molecular weight with MLC visualized by immunoblotting (*middle panel*), in each sample was determined as phosphorylated MLC (p-MLC). The ACh-induced phosphorylation of MLC was augmented in the OA-challenged mice whereas the total MLC levels were equal to the control.

Figure 6 shows the levels of total and phosphorylated MLCs in BSMs determined by immunoblotting and Pro-Q Diamond dye staining, respectively. Immunoblotting with the antibody against MLC protein revealed a single 20 kD band, which contains both phosphorylated and non-phosphorylated MLC proteins (total MLC). The levels of total MLC were the same between groups (Fig. 6, *middle panel*). In the Pro-Q Diamond dye-stained gels, there were several positive bands, *i.e*., phosphorylated proteins [24], in the ACh-stimulated BSM samples. Among them, a 20 kD band corresponding to MLC was distinctly found (Fig. 6, *bottom panel*). The ACh-induced phosphorylation of MLC in BSMs of OA-challenged mice was markedly augmented as compared with those of control animals. A Pro-Q Diamond dye-positive 140 kD band probably corresponding to MYPT1, *i.e*., phosphorylated MYPT1, was also found in the ACh-stimulated BSM samples and was increased in the OA-challenged group (data not shown).

## Discussion

An *in vivo *AHR accompanied by increased IgE production and pulmonary eosinophilia has been demonstrated in the actively sensitized and repeatedly OA-challenged BALB/c strain of mice [20]. By using the same sensitization and challenge protocol in BALB/c mice, the current study demonstrated an increased BSM contractility in ACh-stimulated, but not in high K^+^-depolarized (without receptors stimulation), intact muscle strips of the repeatedly OA-challenged mice (Fig. 1). Likewise, the ACh-induced, C3-sensitive Ca^2+ ^sensitization of BSM contraction was significantly augmented in α-toxin-permeabilized BSMs of the OA-challenged mice (Figs. 2 and 3), whereas the contraction induced by Ca^2+ ^itself was the same as the control level (see Results section). These findings suggest that the C3-sensitive, RhoA-mediated Ca^2+ ^sensitization might be augmented in BSMs of the OA-challenged AHR mice. Indeed, the current study also demonstrated a marked increase in the expression and activation of RhoA protein in BSMs of the AHR mice (Fig. 4 and 5).

In the present study, no significant difference in the Ca^2+^-induced contraction (in the absence of ACh and GTP) of α-toxin-permeabilized BSMs was observed between groups (see Result section), indicating that the contents of typical contractile elements such as calmodulin, myosin light chain (MLC; Fig. 6) and SM α-actin might be the same as control even in the BSMs of the OA-challenged mice. Moreover, the results also indicate that the downstream signaling activated by Ca^2+^-calmodulin complex, including phosphorylation of MLC via activation of MLC kinase, might be in an analogous fashion between groups. The results that the contractile response of intact (non-permeabilized) BSMs induced by high K^+ ^depolarization was not changed after OA challenge also support our speculation. Thus, the baseline Ca^2+ ^sensitivity of contractile elements themselves in BSM cells is unlikely to change in AHR.

By contrast with the contraction induced by Ca^2+ ^itself, the ACh-stimulated contraction of intact BSM strips from the OA-challenged mice was significantly augmented as compared to that from the sensitized control animals (Fig. 1). BSMs are predominantly innervated by vagal efferent nerves, which release ACh when stimulated leading to an activation of muscarinic ACh receptors. The activation of muscarinic receptors existing on BSM, which are mainly thought to be of the M_3 _subtype [27], results in BSM contraction by increasing intracellular Ca^2+ ^concentration through Ca^2+ ^release from sarcoplasmic reticulum and Ca^2+ ^influx from voltage-dependent (nicardipine-sensitive) and receptor-operated (nicardipine-insensitive) Ca^2+ ^channels [28]. Therefore, one possible explanation for the increased response to ACh of OA-challenged BSMs may be attributable to an enhanced Ca^2+ ^mobilization in BSM cells. However, the possibility might be denied by the current result that the ACh-induced contraction of α-toxin-permeabilized BSMs from the OA-challenged mice was significantly augmented as compared with that from the control animals even at a constant Ca^2+ ^concentration (pCa 6.0; Fig. 2B). Moreover, it has also been reported that there is no difference between normal and antigen-induced AHR animals in ACh-induced increase in intracellular Ca^2+ ^concentration in BSMs, irrespective of a great difference in ACh-induced BSM contraction [29,30].

In addition to the classical Ca^2+^-mediated contractile signaling in smooth muscle, it has been demonstrated that agonist stimulation increases myofilament Ca^2+ ^sensitivity in various types of smooth muscles including airways [8,10,21,31]. Recent studies suggest a participation of RhoA in the agonist-induced Ca^2+ ^sensitization of smooth muscle contraction [10]. Hirata *et al*. [32] firstly reported an involvement of RhoA in the mechanism for the increase in Ca^2+ ^sensitization in smooth muscle. It was then shown that RhoA is responsible for the inhibition of MLC phosphatase through the activation of Rho-associated kinases [33]. The present study demonstrated an ACh-induced Ca^2+ ^sensitization in murine BSM contraction (Fig. 2),which is sensitive to C3 exoenzyme (Fig. 3), in the α-toxin-permeabilized BSMs. Furthermore, western blot analysis clearly demonstrated a distinct expression of RhoA protein in BSMs of mice (Fig. 4). Collectively, these findings firstly demonstrated a participation of RhoA-mediated Ca^2+ ^sensitization in ACh-induced BSM contraction in mice.

One of the important findings in the present study is that the C3-sensitive, RhoA-mediated Ca^2+ ^sensitization in ACh-induced contraction was significantly augmented in BSMs of the repeatedly OA-challenged AHR mice (Figs. 2 and 3). Moreover, the protein level of RhoA in BSMs of the AHR mice was significantly increased (Fig. 4). Thus, the current study demonstrated an augmentation of ACh-induced, RhoA-mediated Ca^2+ ^sensitization of BSM contraction, which coincides with enhanced protein expression of RhoA, in antigen-induced AHR. Although the mechanism(s) of up-regulation of RhoA in OA-challenged BSMs is not known here, inflammatory cytokines such as tumor necrosis factor-α [34], which is also demonstrated in airways of this murine model of asthma (unpublished data), may be involved in. On the other hand, it has been reported that an introduction of active forms of RhoA to permeabilized smooth muscle induced contractile response [32,35]. It is thus likely that ACh stimulation activates the upregulated RhoA (Fig. 5), resulting in a greater phosphorylation of MLC (Fig. 6) and contraction of BSMs in AHR mice.

An increase in responsiveness to muscarinic agonists of airway smooth muscle has been demonstrated in animal models of AHR [21,22,36,37] and asthmatic patients [38], although no change in the levels of plasma membrane receptors was observed [36,37,39]. Moreover, the agonist-induced increase in cytosolic Ca^2+ ^level was within normal level even in the hyperresponsive BSMs [29,30]. Taken together with our current findings, it is likely that the enhanced contractility to agonists reflects, at least in part, the augmentation of muscarinic receptor- and RhoA-mediated Ca^2+ ^sensitization, although the mechanism(s) for activation of RhoA by ACh is still unclear. If RhoA proteins are activated by receptors other than muscarinic receptor, it might account for the 'non-specific' AHR, which is a common feature of allergic asthmatics. Indeed, the BSMs of the OA-challenged mice also have hyperresponsiveness to endothelin-1 [40], which has been reported to activate RhoA via its own receptors [41].

An upregulation of RhoA/Rho-kinase associated with the augmented smooth muscle contractility has also been reported in rat myomertium during pregnancy [42,43], arterial smooth muscle of spontaneously hypertensive rats [12], coronary vasospasm in pigs [16], dog femoral artery in heart failure [44], and BSMs in rat experimental asthma [21]. Thus, the upregulation of RhoA might be widely involved in the enhanced contraction of the diseased smooth muscles including the BSMs in AHR over species.

## Conclusions

In conclusion, the current study demonstrated an ACh-induced, RhoA-mediated Ca^2+ ^sensitization in murine BSM contraction. An augmentation of the Ca^2+ ^sensitizing effect, probably by the upregulation of RhoA protein, might be involved in the enhanced BSM contraction observed in the antigen-induced AHR in mice.

## Authors' contributions

YC conceived of the study, participated in its design and coordination, and drafted the manuscript. AU carried out the intact smooth muscle studies. KS, HT and HS carried out the skinned fiber studies and immunoblot analysis. SN carried out the analysis of active RhoA. MM participated in the direction of the study as well as writing and preparing the manuscript. All authors read and approved the final manuscript.
